# The impact of mobile demersal fishing on carbon storage in seabed sediments

**DOI:** 10.1111/gcb.16105

**Published:** 2022-02-17

**Authors:** Graham Epstein, Jack J. Middelburg, Julie P. Hawkins, Catrin R. Norris, Callum M. Roberts

**Affiliations:** ^1^ 3286 Centre for Ecology and Conservation University of Exeter Cornwall UK; ^2^ Department of Earth Sciences Utrecht University Utrecht The Netherlands

**Keywords:** blue carbon, carbon, carbon storage, dredging, fishing, marine, sediment, trawling

## Abstract

Subtidal marine sediments are one of the planet's primary carbon stores and strongly influence the oceanic sink for atmospheric CO_2_. By far the most widespread human activity occurring on the seabed is bottom trawling/dredging for fish and shellfish. A global first‐order estimate suggested mobile demersal fishing activities may cause 0.16–0.4 Gt of organic carbon (OC) to be remineralized annually from seabed sediment carbon stores (Sala et al., 2021). There are, however, many uncertainties in this calculation. Here, we discuss the potential drivers of change in seabed sediment OC stores due to mobile demersal fishing activities and conduct a literature review, synthesizing studies where this interaction has been directly investigated. Under certain environmental settings, we hypothesize that mobile demersal fishing would reduce OC in seabed stores due to lower production of flora and fauna, the loss of fine flocculent material, increased sediment resuspension, mixing and transport and increased oxygen exposure. Reductions would be offset to varying extents by reduced faunal bioturbation and community respiration, increased off‐shelf transport and increases in primary production from the resuspension of nutrients. Studies which directly investigated the impact of demersal fishing on OC stocks had mixed results. A finding of no significant effect was reported in 61% of 49 investigations; 29% reported lower OC due to fishing activities, with 10% reporting higher OC. In relation to remineralization rates within the seabed, four investigations reported that demersal fishing activities decreased remineralization, with three reporting higher remineralization rates. Patterns in the environmental and experimental characteristics between different outcomes were largely indistinct. More evidence is urgently needed to accurately quantify the impact of anthropogenic physical disturbance on seabed carbon in different environmental settings and to incorporate full evidence‐based carbon considerations into global seabed management.

## Graphical abstract

Subtidal marine sediments are one of the planet's primary carbon stores and strongly influence the oceanic sink for atmospheric CO_2_. Considering basic principles, we hypothesize that under certain environmental settings, trawling/dredging for fish and shellfish would reduce seabed sediment carbon storage; however, this may be offset by positive feedback mechanisms in some circumstances. A review of studies which directly investigated this impact in situ also highlighted mixed results. More evidence is urgently needed to accurately quantify this impact in different environmental settings, and incorporate full evidence‐based carbon considerations into global seabed management.

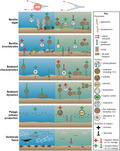

## 1 INTRODUCTION

Through a mixture of physical, chemical and biological processes, the ocean has absorbed ~40% of anthropogenic CO_2_ emissions since the industrial revolution (Gruber et al., 2019; Sabine & Tanhua, 2010). The term ‘blue carbon’ describes the ability of marine ecosystems to absorb CO_2_ from the atmosphere or water column, assimilate this inorganic carbon (IC) into organic compounds and isolate it from remineralization for centennial to millennial timescales (Nellemann et al., 2009). This process of carbon capture is key to maintaining the ecological functioning of the ocean (Bauer et al., 2013) and is beneficial as a sink for anthropogenic CO_2_ (Gruber et al., 2019; Khatiwala et al., 2009; Watson et al., 2020).

Research on blue carbon initially focused on the coastal vegetated habitats of mangroves, seagrass and saltmarsh, due to their ability to fix CO_2_ directly, trap external organic and inorganic materials, store high concentrations of organic carbon (OC) in situ within underlying sediments and accrete at high rates (Duarte et al., 2013; McLeod et al., 2011). Although these habitats have among the highest carbon sequestration rates on the planet per unit area (Duarte et al., 2013), with rates considerably higher than forests on land (McLeod et al., 2011), their limited spatial scale of approximately 1 million km^2^ or ~0.2% of the ocean's surface means they only contain a small proportion of the ocean's total OC stock (Atwood et al., 2020; Duarte, 2017; Duarte et al., 2013; Howard et al., 2017; Macreadie et al., 2021; Nellemann et al., 2009).

Primary production by phytoplankton is at par with terrestrial primary production (Field et al., 1998) and constitutes a major flux between the oceanic IC pool, hence the atmosphere, and the oceanic OC pool (Sarmiento & Gruber, 2006). A variable fraction of this newly produced OC is exported to depths below 1000 m, where this pelagic OC may become isolated from atmospheric exchange processes for centennial timescales (Caldeira et al., 2002; Krause‐Jensen & Duarte, 2016; Nellemann et al., 2009; Siegel et al., 2021). Whether this pelagic OC stock can be better protected or enhanced, and the duration of time for which it remains isolated from atmospheric exchange under different environmental settings is still uncertain. Pelagic OC is therefore infrequently used in the quantification of marine carbon storage and rarely classified as blue carbon (Caldeira et al., 2002; Lovelock & Duarte, 2019; Siegel et al., 2021). That withstanding subtidal marine sediments contain the ocean's biggest OC store, estimated to hold ~87 Gt of OC (1 Gt = 1 Pg = 10^15^ g) in the upper 5 cm (Lee et al., 2019) or ~2300 Gt of OC in the top 1 m (Atwood et al., 2020). Quantification of annual burial rates in these sediments is poorly constrained; however, they have been estimated globally at approximately 0.12–0.35 Gt OC year^−1^ (Berner, 1982; Burdige, 2007; Keil, 2017; Lee et al., 2019; Seiter et al., 2004).

Seabed sediments are subjected to a wide range of direct physical impacts from human pressures, namely, shipping, mineral extraction, fishing, energy developments, deployment of cables and pipelines, coastal development, dredging of shipping access channels and disposal of dredge spoil (Halpern et al., 2019; O’Hara et al., 2021). By far the most widespread source of disturbance is bottom trawling and dredging for fish and shellfish (Amoroso et al., 2018; Eigaard et al., 2017; Kroodsma et al., 2018; O’Hara et al., 2021; Oberle, Storlazzi, & Hanebuth, 2016). These pressures are pervasive and long‐lasting, with improved technologies over the last two centuries, and in particular since the 1950s, increasing the spread of mobile fishing gears to deeper waters and much of the global ocean (Kroodsma et al., 2018; Morato et al., 2006; Roberts, 2007; Watson & Morato, 2013). Compared to many other types of stressors, in intensively fished areas, trawling and dredging can also occur on the same area of seabed numerous times in a year (Eigaard et al., 2017; Hinz et al., 2009; Oberle, Storlazzi, & Hanebuth, 2016; Tillin et al., 2006).

Globally, fishing pressure with mobile demersal gear is concentrated in subtidal areas in coastal habitats and offshore on continental shelves and slopes at depths above 1000 m (Amoroso et al., 2018; Kroodsma et al., 2018). In total, these areas cover around 9% of the global seabed, yet they store an estimated 360 Gt OC in their top 1 m of sediment (Atwood et al., 2020) and are estimated to account for up to 86% of all OC that is buried annually in global subtidal sediments (Atwood et al., 2020; Berner, 1982; Seiter et al., 2004).

Mobile demersal fishing activity significantly alters seabed faunal communities (Hiddink et al., 2017; Kaiser et al., 2006; Sciberras et al., 2016), restructures the top layers of benthic sediments (Eigaard et al., 2016; Oberle, Swarzenski, et al., 2016; Puig et al., 2012; Trimmer et al., 2005) and resuspends large volumes of sediment into the water column (de Madron et al., 2005; Jones, 1992; Martín, Puig, Palanques, & Giamportone, 2014; Palanques et al., 2014; Ruffin, 1998; Thrush & Dayton, 2002). However, the net effect of this disturbance on OC stores is poorly resolved. Through mixing, resuspension and oxidation of surface sediments, along with the disturbance of benthic communities, fishing may generate a source of ‘underwater carbon dioxide emissions’ via increased remineralization of OC and may also limit future OC burial by inhibiting long‐term sediment settlement and consolidation (De Borger et al., 2021; Keil, 2017; Luisetti et al., 2019; Martín, Puig, Palanques, & Giamportone, 2014; Sala et al., 2021). This disturbance may increase IC concentrations in the ocean, lower its buffering capacity and via this, slow the rate of CO_2_ uptake from the atmosphere, while contributing to ocean acidification and potentially leading to increased release of oceanic CO_2_ to the atmosphere (Bauer et al., 2013; Keil, 2017; Khatiwala et al., 2009; LaRowe et al., 2020; Lovelock et al., 2017; Luisetti et al., 2019; Pendleton et al., 2012; Sala et al., 2021). However, to place the effect of mobile demersal fishing in full context, it is important to better quantify the impacts of different pressures on OC storage and to understand how these compare with natural hydrological disturbances to seabed sediments in different environmental settings (Arndt et al., 2013; Pusceddu et al., 2005; Rühl et al., 2020; Winterwerp & Kranenburg, 2002).

The cycling and storage of OC at the seabed is highly complex and is influenced by sediment fauna, flora and microbes; seabed lithology and granulometry; and the chemistry, hydrology and biology of the surrounding water column (Bauer et al., 2013; Burdige, 2007; Keil, 2017; LaRowe et al., 2020; Middelburg, 2018; Rühl et al., 2020; Snelgrove et al., 2018). With all of these factors affected by many positive and negative feedback mechanisms, it is challenging to definitively identify the impact of trawling and dredging on net OC storage (Keil, 2017; LaRowe et al., 2020; Rühl et al., 2020; Snelgrove et al., 2018). In this review, we discuss the potential drivers of change in sediment OC due to mobile demersal fishing activities and summarize empirical evidence where their effects on sediment OC have been directly investigated. We also discuss recent peer‐reviewed publications which aim to quantify the impact of mobile demersal fishing at global, regional and national scales, and highlight why the results must be viewed with concern and caution (Luisetti et al., 2019; Paradis et al., 2021; Sala et al., 2021). If seabed sediments were to be recognized as a quantifiable and manageable blue carbon resource, it could unlock huge climate change mitigation potential and carbon financing opportunities (Avelar et al., 2017; Seddon et al., 2019).

## 2 LINKS BETWEEN SEABED SEDIMENT OC AND MOBILE DEMERSAL FISHING

### 2.1 Production of benthic micro‐ and macroalgae

Seabed sediment OC is mostly allochthonous, with much of it originating from terrestrial run‐off and primary production in surface waters from phytoplankton, macroalgae and wetland vegetation (Bauer et al., 2013; Krause‐Jensen & Duarte, 2016; LaRowe et al., 2020; Legge et al., 2020; Turner, 2015). Much of this OC will be consumed, repackaged, excreted or remineralized before a small remaining proportion of OC reaches the seabed (Keil, 2017; Middelburg, 2018; Turner, 2015). On sediments in the euphotic zone, some OC is autochthonous—that is, produced in situ by microphytobenthos, and by macroalgae found on more stable sediments, hard substrate or attached to biogenic material (Gattuso et al., 2006; MacIntyre et al., 1996).

**FIGURE 1:**
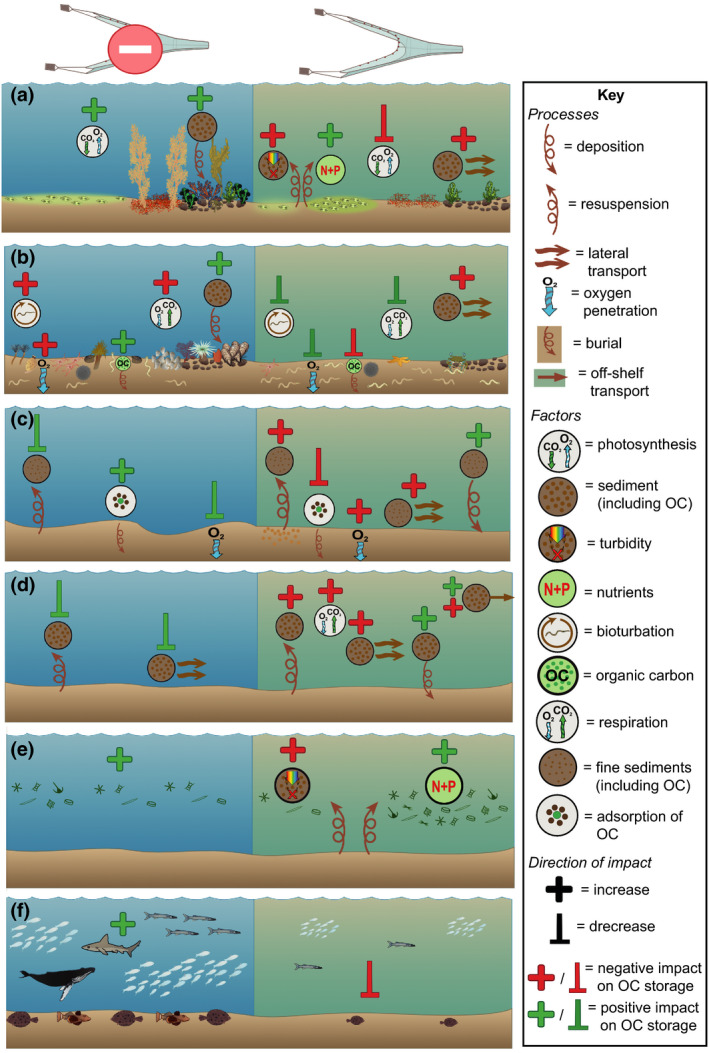
Potential impact of mobile demersal fishing on processes that affect seabed sediment OC (organic carbon) storage. The effects of mobile demersal fishing activity (right) and absence of demersal fishing activity (left) are shown on: (a) benthic algae, (b) benthic infauna and epifauna, (c) sediment characteristics, (d) sediment dynamics, (e) pelagic primary production, (f) vertebrate fauna and how each of these changes may impact OC storage. Addition symbols indicate when a factor/process would be expected to increase in the presence/absence of fishing whereby inhibitory arrows indicate when a factor/process would be expected to decrease. The colour of the addition/inhibition symbols indicates whether this change is predicted to impact OC sequestration and storage either positively (green) or negatively (red). Symbols courtesy of Integration and Application Network (ian.umces.edu/media‐library)

While the impact of mobile demersal fishing on benthic algae is little studied, it is known that benthic macroalgae are easily damaged by physical disturbance, and the structure and abundance of microphytobenthos are highly dependent on both natural and anthropogenic perturbation (Fragkopoulou et al., 2021; Larson & Sundbäck, 2012; MacIntyre et al., 1996). At least in the short term, mobile demersal fishing can reduce algal cover and sediment surface chlorophyll *a* concentration (Figure 1a) (Fragkopoulou et al., 2021; MacIntyre et al., 1996; Mayer et al., 1991; Tiano et al., 2019; Watling et al., 2001). For example, scallop dredging at depths of 8–15 m in the Damariscotta River Estuary of the Northwest Atlantic led to clear visual disturbance of diatom mats and caused a significant reduction in chlorophyll *a* concentration (Mayer et al., 1991; Watling et al., 2001). However, there are mixed results in some longer term studies (Brylinsky et al., 1994; Pusceddu et al., 2014).

Among algae, kelp and coralline algae can require years and decades, respectively, to recover following disturbance (e.g. Dayton et al., 1992; Fragkopoulou et al., 2021). By contrast, ephemeral macroalgae and microphytobenthos can recover quickly, especially from less chronic disturbance (MacIntyre et al., 1996; Ordines et al., 2017). For example, in the *Pesquera Rica* trawling grounds of the Balearic Islands, red algae beds of Peyssonneliaceae and Corallinophycidae persist within trawled areas, although their biomass is around 39%–47% lower compared to untrawled areas (Ordines et al., 2017).

As well as physically impacting the benthos, disturbance from mobile demersal fishing also releases nutrients from subsurface sediments which may promote primary production in benthic algae, especially in oligotrophic environments (Figure 1a) (Dounas et al., 2007; Falcão et al., 2003; Fanning et al., 1982). Dependent on the environmental setting, this may offset some of the losses from direct physical disturbance. However, counteracting this, sediment suspended by fishing can increase turbidity (Capuzzo et al., 2015; Palanques et al., 2001; Ruffin, 1998) which reduces light penetration and thus photosynthetic rates (Figure 1a) (MacIntyre et al., 1996).

In many settings, high‐frequency mobile demersal fishing would be expected to reduce the abundance of benthic flora on euphotic sediments and is therefore predicted to limit the OC supply and quantity stored directly, and via secondary production (Figure 1a) (Mandal et al., 2021; Middelburg, 2018; Miller et al., 1996). Additionally, benthic micro‐ and macroalgae are known to increase the stability and accumulation rate of seabed sediments (Miller et al., 1996; Montserrat et al., 2008; Yallop et al., 1994), a primary driver of OC burial and storage (LaRowe et al., 2020; Middelburg, 2018). This represents a further mechanism through which the disturbance of benthic algae from mobile demersal fishing could limit the potential burial rate of OC within sedimentary seabed habitats (Figure 1a).

### 2.2 Benthic faunal production and processing of OC

The impact of mobile demersal fishing gears on benthic fauna has been widely studied. It depends on the intensity, depth and frequency of demersal fishing; legacy of prior perturbations; type of fishing gear; intensity and frequency of natural disturbances; sediment type; and benthic community composition; and is consequently site‐specific (Collie et al., 2000; Hiddink et al., 2017; Kaiser et al., 2002, 2006; Sciberras et al., 2018; Thrush & Dayton, 2002). Gears which penetrate most deeply into sediment, such as dredges and hydraulic gears, tend to have greater impact than gears with less penetration, such as demersal seines and otter trawls (Collie et al., 2000; Hiddink et al., 2017; Kaiser et al., 2006; Sciberras et al., 2018), although habitat type also has an influence (Rijnsdorp et al., 2020). The largest impacts follow initial experimental trawling events or are seen when comparisons are made to an area of long‐standing protection (Cook et al., 2013; Thrush & Dayton, 2002). Many studies may underestimate the damage done by mobile fishing gears and overestimate the speed of recovery because they measure the recovery of areas already impacted (Collie et al., 2000; Cook et al., 2013; Hiddink et al., 2017; Hinz et al., 2009; Kaiser et al., 2002, 2006; Sciberras et al., 2018).

To a greater or lesser extent, bottom trawling and dredging reduce total benthic biomass and production of benthic macrofauna, and cause loss in abundance and diversity of sessile epifauna and long‐lived shallow burrowing infauna (Jennings et al., 2001, 2002; Kaiser et al., 2002; Queirós et al., 2006; Sciberras et al., 2018; Tiano et al., 2020; Tillin et al., 2006). Long‐term fishing with mobile gears leads to the preponderance of small‐bodied, opportunistic, motile infauna, and larger, highly vagrant, scavenging macrofauna (Jennings et al., 2001, 2002; Kaiser et al., 2002, 2006; Thrush & Dayton, 2002; Tillin et al., 2006). Mobile demersal fishing can also directly affect the diversity and community structure within the largely resistant opportunistic meiofauna (Pusceddu et al., 2014; Schratzberger et al., 2009).

Benthic fauna are strong drivers of biogeochemical cycling in sediments (LaRowe et al., 2020; Middelburg, 2018; Rühl et al., 2020; Snelgrove et al., 2018). For example, in a well‐studied area off the coast of Vancouver Island, taxonomic and functional richness of benthic fauna explained a similar proportion of variance in pelagic–benthic nutrient flux (~20%) when compared to a suite of environmental variables (Belley & Snelgrove, 2016, 2017). OC that reaches the seabed is directly consumed by deposit and suspension feeding fauna, and is thereafter incorporated into biomass, expelled as faeces and pseudofaeces or metabolized and remineralized through respiration (Arndt et al., 2013; Keil, 2017; Middelburg, 2018; Rühl et al., 2020; Snelgrove et al., 2018). While respiration reduces the total amount of OC available for burial and storage, the preferential utilization of labile OC by benthic communities may result in the accumulation of refractory compounds that are more resistant to microbial decomposition (Figure 1b) (Arndt et al., 2013; LaRowe et al., 2020; Middelburg, 2018).

Bioturbation and bio‐irrigation are, respectively, the reworking of sediment particles and solutes by fauna (Ekdale et al., 1984; Meysman et al., 2006); this can impact OC remineralization in two ways. First, bio‐irrigation activities enhance the oxygenation of surface sediments and cause an increase in the concentration of other electron acceptors such as nitrate, metal oxides and sulphate, therefore promoting microbial degradation of OC (Figure 1b) (Arndt et al., 2013; Hulthe et al., 1998; Keil, 2017; LaRowe et al., 2020; Meysman et al., 2006; Snelgrove et al., 2018). Second, particle mixing transports labile OC from the surface to deeper sediment layers. On the one hand, this potentially increases their chance of burial and long‐term storage (Figure 1b) (De Borger et al., 2021; Middelburg, 2018; Rühl et al., 2020; Snelgrove et al., 2018; van der Molen et al., 2012). On the other hand, the transfer of high‐quality OC from the surface to deeper layers may prime microbial communities and in this way stimulate degradation of more refractory OC found in deeper sediment layers (Middelburg, 2018; van Nugteren et al., 2009). This can lead to significantly increased total OC remineralization rates, although the process is known to vary between environmental settings (Bengtsson et al., 2018; Riekenberg et al., 2020; van Nugteren et al., 2009).

The composition and abundance of benthic fauna can also influence the stability and accumulation rates of sediment, which are key drivers of OC burial and storage (LaRowe et al., 2020; Middelburg, 2018). While increased bioturbation activity generally has a destabilizing effect, burrowing fauna can increase the stability and accumulation rate of sediment if there is an increase in biogenic materials such as worm tubes or mucus production, or an increase in structural complexity at the sediment surface from the presence of sedentary and sessile epifauna and biogenic habitat (Figure 1b) (Borsje et al., 2014; Ekdale et al., 1984; Roberts, 2007; Rühl et al., 2020; Thrush & Dayton, 2002). For example, in fine sands and muds of the Northeast Atlantic, the presence of the tube building polychaete *Lanice conchilega* can lead to increased sediment accretion rates due to changes in flow dynamics around the worm tubes, with impacts on sedimentation dynamics beyond the biogenic structure and over a longer duration than the lifetime of an individual worm (Borsje et al., 2014). As species that build biogenic structures are particularly vulnerable to damage from mobile demersal fishing (Fariñas‐Franco et al., 2018; Kaiser et al., 2002), their abundance and distribution could be greatly altered by the widespread nature of this pressure.

Faunal biomass and production are some of the main contributors of OC in seabed sediments; therefore, in many environmental settings, the impact of mobile demersal fishing on benthic fauna is hypothesized to cause a reduction in seabed OC storage. However, this effect would be offset to varying extents by reduced bioturbation, OC consumption and respiration causing lower remineralization rates. The balance depends on the many complex interactions discussed above, which are site‐specific.

### 2.3 Alteration to sediment composition

Mobile demersal fishing gears can alter the granulometry, topography and vertical structuring of seabed sediments (Depestele et al., 2019; Martín, Puig, Palanques, & Giamportone, 2014; Oberle, Storlazzi, & Hanebuth, 2016; Oberle, Swarzenski et al., 2016; Puig et al., 2012; Trimmer et al., 2005), with extent of change influenced by gear used, sediment type, local hydrology and frequency of fishing (Kaiser et al., 2002; Martín, Puig, Palanques, & Giamportone, 2014; Oberle, Swarzenski et al., 2016; O'Neill & Summerbell, 2016; O'Neill et al., 2018; Trimmer et al., 2005). Gears that penetrate more deeply into sediment and have a larger footprint cause most impact (Depestele et al., 2015, 2019; Eigaard et al., 2016; Kaiser et al., 2002; Martín, Puig, Palanques, & Giamportone, 2014). In highly mobile habitats, for example, ones with abundant shallow sand, the structure and composition of sediment may not be greatly altered by mobile demersal fishing due to strong natural forcing mechanisms, while those found in less hydrologically active environments could be highly affected (Kaiser et al., 2002; Martín, Puig, Palanques, & Giamportone, 2014; Oberle, Swarzenski et al., 2016; Trimmer et al., 2005). However, greater sediment mobility may itself be a consequence of long‐term use of mobile fishing gears, due to loss of fauna and flora that stabilize sediments (Roberts, 2007).

Topographic alterations from mobile fishing gears can consist of visible trawl/dredge tracks and homogenization in large‐scale seabed topography (Depestele et al., 2015, 2019; Eigaard et al., 2016; Kaiser et al., 2002; Martín, Puig, Palanques, & Giamportone, 2014; Oberle, Storlazzi, & Hanebuth, 2016; Oberle, Swarzenski, et al., 2016; O'Neill & Summerbell, 2016; Palanques et al., 2014; Tiano et al., 2020). For example, multibeam surveys have shown that chronic trawling on the continental slopes of the Palamós canyon in the Northwest Mediterranean has had drastic flattening effects on soft sediments (Puig et al., 2012). Mobile demersal fishing also mixes and overturns the top layer of seabed, generally causing a homogenization of the sediment structure and an increase in density of surface sediments (Depestele et al., 2019; Martín, Puig, Masque, et al., 2014; Oberle, Swarzenski, et al., 2016; Paradis et al., 2021; Pusceddu et al., 2014). The sediment's vertical profile can also be altered, with an increase in coarse material towards the surface, caused by winnowing, resuspension and loss of fine material (Figure 1c) (Martín, Puig, Masque, et al., 2014; Martín, Puig, Palanques, & Giamportone, 2014; Mengual et al., 2016; Oberle, Swarzenski, 2016; Palanques et al., 2014; Paradis et al., 2021; Pusceddu et al., 2014). If the local hydrology favours deposition, the sediment may be overlain by a surface layer of fine material from the redeposition of fine sediment which has been resuspended from deeper layers (Oberle, Swarzenski, et al., 2016; Palanques et al., 2014; Tiano et al., 2020). On the Northwest Iberian shelf, all these processes and impacts were identified within a study across different trawling intensities and environmental settings, highlighting the complexity in predicting fine‐scale effects of mobile demersal fishing on sediment structure (Oberle, Swarzenski, et al., 2016).

The physical mixing of surface sediments can cause an increase in oxygen penetration and/or sediment oxygen concentrations (Allen & Clarke, 2007; De Borger et al., 2021; Tiano et al., 2019). Although oxygen penetration depths usually rapidly reestablish after perturbations, they do not necessarily return to a predisturbed state (Allen & Clarke, 2007; De Borger et al., 2021; Tiano et al., 2019). Increased sediment oxygenation can increase microbial respiration and remineralization of OC (Figure 1c) (Dauwe et al., 2001; Keil, 2017; Kristensen et al., 1995; van de Velde et al., 2018). Mixing of sediments by mobile demersal fishing may also transport OC from the surface to deeper sediment layers (Mayer et al., 1991). As with faunal‐mediated sediment reworking, this may increase the chance of burial and long‐term storage, but may also stimulate the degradation of more refractory OC that was already present in deeper sediment layers (Duplisea et al., 2001; Mayer et al., 1991; Middelburg, 2018; van Nugteren et al., 2009).

The loss of fine, flocculent material and OC–mineral interactions is another mechanism by which OC storage could be reduced (Figure 1c) (Martín, Puig, Palanques, & Giamportone, 2014; Oberle, Storlazzi, & Hanebuth, 2016; Pusceddu et al., 2014). The process of physical encapsulation of OC by sediment particles and the resultant protection from remineralization is seen as a key process in long‐term OC storage (Arndt et al., 2013; Burdige, 2007; Estes et al., 2019; Hemingway et al., 2019; LaRowe et al., 2020). For example, in sediment samples from the Northeast Pacific coasts of Mexico and Washington state, 50% of the oldest OC stores were sorbed to mineral surfaces (Arnarson & Keil, 2007). Fine‐grained sediments such as silts and clays with large specific surface areas and often reduced redox potentials typically have higher OC contents compared to habitats dominated by sand and coarse sediment (Burdige, 2007; Paradis et al., 2021; Smeaton et al., 2021). As mobile demersal fishing generally exposes or suspends fine material, this may reduce overall OC storage through loss of OC–mineral interactions and remineralization (Figure 1c) (Arnarson & Keil, 2007; Estes et al., 2019). However, if disturbed OC associated with fine sediments is largely refractory in nature and is not remineralized but reallocated to surface sediments in situ or transported laterally to different locations, this disturbance may increase seabed surface OC in certain environmental settings (Figure 1c) (LaRowe et al., 2020; Oberle, Swarzenski, et al., 2016; Palanques et al., 2014; Tiano et al., 2021).

### 2.4 Sediment resuspension and transport

Large volumes of seabed sediments can be moved laterally and vertically, and become resuspended in the water column by tides, waves and storms (Ferré et al., 2008; Soulsby, 1997; Winterwerp & Kranenburg, 2002). Mobile demersal fishing activities are a large contributor to the quantities of sediment displaced by these natural forcing mechanisms (Depestele et al., 2015; Ferré et al., 2008; Jones, 1992; Martín, Puig, Palanques, & Giamportone, 2014; Mengual et al., 2016; Oberle, Storlazzi, & Hanebuth, 2016; O'Neill & Summerbell, 2016; Paradis et al., 2018; Pusceddu et al., 2005, 2015). Magnitudes involved are highly dependent on depth, gear and sediment type, with deeper penetrating gears and finer sediments causing larger dispersed volumes (Churchill, 1989; de Madron et al., 2005; Ferré et al., 2008; Martín, Puig, Palanques, & Giamportone, 2014; Mengual et al., 2016; Oberle, Storlazzi, & Hanebuth, 2016; O'Neill & Ivanović, 2015; O'Neill & Summerbell, 2016; Palanques et al., 2014; Pusceddu et al., 2005; Ruffin, 1998). Depending on local hydrographic conditions, sediment may remain in suspension for extended periods of time, and can be transported across large vertical and lateral distances (de Madron et al., 2005; Ferré et al., 2008; Martín et al., 2006, 2008, 2014; Oberle, Storlazzi, & Hanebuth, 2016; Palanques et al., 2006, 2014; Pusceddu et al., 2015). In the Northern Mediterranean, otter trawling resulted in average suspended sediment concentrations ranging between 6 and 50 mg/L, depending on the study site (de Madron et al., 2005; Palanques et al., 2001). Sediment within the water column was found to persist for up to 5 days (Palanques et al., 2001), while off‐shelf transport was 1.4–9 times higher when compared to sediment volumes without trawling (Ferré et al., 2008; Palanques et al., 2014). The loss of seabed topography, as discussed above (Martín, Puig, Palanques, & Giamportone, 2014; Oberle, Swarzenski, et al., 2016; Puig et al., 2012), may also alter local‐scale hydrographic conditions, increasing sediment boundary water flows and the magnitude of sediment resuspension (Smith & McLean, 1977; Soulsby, 1997).

Natural sediment disturbance during storms is known to stimulate increased water column microbial production (Cotner et al., 2000) and OC remineralization rates (Pusceddu et al., 2005; Wainright & Hopkinson Jr, 1997). The resuspension and transport of sediment from mobile demersal fishing is hypothesized to lead to a reduction in OC concentration (Martín, Puig, Palanques, & Giamportone, 2014), largely due to increased oxygen exposure times and shifts between anoxic and oxic states, which can increase remineralization rates (Figure 1d) (Dauwe et al., 2001; Hulthe et al., 1998; Keil, 2017; Kristensen et al., 1995). Fishing‐induced disturbance may further promote remineralization, as sediment which is deposited under oxic conditions, then buried under anoxia and re‐exposed to oxygen can stimulate OC degradation rates (Hulthe et al., 1998). This has been identified in the biochemical signature of suspended particulate OC within trawling grounds of the North Mediterranean, with a significant shift from labile to refractory OC compounds (Pusceddu et al., 2005, 2005, 2015).

Previous studies have shown that it is challenging to fully quantify the amount of OC that will be remineralized after disturbance, rather than simply being moved elsewhere (Lovelock et al., 2017; Martín et al., 2006, 2008; Pusceddu et al., 2005; Wainright & Hopkinson Jr, 1997). There is also the potential that sediment resuspension from mobile demersal fishing could increase OC storage in adjacent areas (Figure 1d). This could occur from higher sedimentation rates near to fishing grounds leading to increased burial of OC which is already present within the seabed, or burial of benthic algae and sessile fauna (Churchill, 1989; Jones, 1992; Oberle, Storlazzi, & Hanebuth, 2016; Sciberras et al., 2016). It could also lead to the transportation of OC‐rich shelf and slope sediments (Atwood et al., 2020) to deeper waters which may be isolated from atmospheric exchange for centennial timeframes (Figure 1d) (Caldeira et al., 2002; Ferré et al., 2008; Martín et al., 2006, 2008; Paradis et al., 2018; Siegel et al., 2021). Such off‐shelf induced transport of sediment and OC has been recorded as deep as 1750 m in continental slope trawling grounds of the Palamós canyon in the Northwest Mediterranean (Martín et al., 2006, 2008; Palanques et al., 2006). Any OC transport from shelf to deeper waters that are isolated from the atmosphere for centennial timeframes could be considered a sink for atmospheric carbon dioxide, irrespective of whether the carbon accumulates as OC in sediments or is respired to carbon dioxide.

Increased sediment resuspension from mobile demersal fishing is predicted to reduce the current store of OC in seabed sediments due to the disturbance of accumulations and increased oxygen exposure times (Keil, 2017; Luisetti et al., 2019; Martín, Puig, Palanques, & Giamportone, 2014; Sala et al., 2021). Future burial may also be limited as newly settled organic material would be kept in suspension, precluding it from burial and storage (Churchill, 1989; Martín, Puig, Palanques, & Giamportone, 2014; Oberle, Storlazzi, & Hanebuth, 2016; Ruffin, 1998). However, reductions in OC could be offset to varying extents by trawl‐induced burial of OC through sediment mixing, redeposition and increased off‐shelf transport of OC (Martín et al., 2008; Mayer et al., 1991). The site‐specific nature of this impact will be largely based on the vulnerability of OC to remineralization (Arndt et al., 2013; Middelburg, 2018) and local hydrography, which will primarily determine the fate of resuspended OC (Ferré et al., 2008; Keil, 2017; LaRowe et al., 2020; Wainright & Hopkinson Jr, 1997). In highly dynamic environments and where sediment OC is highly refractory, the additional impact of fishing‐related disturbance on sediment OC may be limited.

### 2.5 Alteration in pelagic primary production

As most seabed OC is allochthonous, the total amount which reaches seabed sediments is strongly driven by the level of primary production in the overlying water column (Atwood et al., 2020; Seiter et al., 2004; Turner, 2015). Sediment disturbance by mobile fishing gears, or natural forces, can lead to a decrease or increase in primary production. Resuspension of particles can lead to stronger attenuation of light conditions with the consequence that primary production decreases (Figure 1e) (Adriano et al., 2005; Capuzzo et al., 2015; Cloern et al., 2014; Palanques et al., 2001; Ruffin, 1998). However, sediment disturbance can release significant concentrations of nutrients into the water column (de Madron et al., 2005; Falcão et al., 2003; Fanning et al., 1982; Polymenakou et al., 2005; Pusceddu et al., 2015). In shallower areas, released nutrients will likely enter into or remain in the euphotic zone, where their fertilization effect can increase phytoplankton primary production (Figure 1e) (Dounas et al., 2007; Fanning et al., 1982; Palanques et al., 2014). For example, modelling predictions from trawling experiments in the Eastern Mediterranean at Heraklion Bay led to estimates that nutrient upwelling from bottom trawling could increase net annual primary production by 15% (Dounas et al., 2007) with subsequent settlement raising OC in seabed sediments (Falcão et al., 2003; Palanques et al., 2014; Polymenakou et al., 2005; Turner, 2015). The net effect of demersal fishing activity on pelagic primary production is therefore expected to differ in systems where primary producers are limited by either light or nutrients.

### 2.6 The contribution of vertebrate fauna to OC storage

Although not a focus of this review, the removal of vertebrate species by benthic and pelagic fisheries could influence the mass of OC stored in seabed sediments (Atwood et al., 2015; Mariani et al., 2020; Pershing et al., 2010). The emerging field of ‘fish carbon’ describes the contribution of vertebrate fauna to OC storage within seabed sediments from defecation, pelagic mixing, bioturbation, trophic interactions and deadfall (Saba et al., 2021; Trueman et al., 2014; Turner, 2015). Although the magnitudes of effect are poorly resolved, the reduction in population size and average body size of marine vertebrates that can occur from their exploitation (Britten et al., 2021; Hatton et al., 2021; Pacoureau et al., 2021) is predicted to reduce the amount of carbon exported to the seabed (Figure 1f) (Atwood et al., 2015; Bianchi et al., 2021; Mariani et al., 2020; Pershing et al., 2010; Trueman et al., 2014). For example, since 1950, the combined catch of Tuna, Mackerel, Shark and Billfish is estimated to have prevented approximately 0.02 Gt of OC being stored in seabed sediments (Mariani et al., 2020). The removal of predatory vertebrates will also cause trophic cascades, potentially leading to alterations in benthic faunal communities, triggering the feedback mechanisms on OC discussed above (Atwood et al., 2015). Further research which uses fingerprinting techniques to identify the provenance of OC in seabed sediments under different environmental settings may allow the magnitude of contribution by vertebrate fauna to be better resolved (Geraldi et al., 2019; Larsen et al., 2013); however, processes such as pelagic mixing, bioturbation and trophic interactions would remain challenging to fully quantify.

### 2.7 Interactions and feedback mechanisms

Although largely outside of the scope of this review, the six main factors discussed here interact in a variety of positive and negative feedback loops which will add further complexity to outcomes on seabed sediment OC. For example, the alterations in sediment structure that may occur due to mobile demersal fishing activities will, in itself, influence the community structure of benthic flora and fauna even if the biota are not significantly impacted by the physical disturbance itself (McArthur et al., 2010). A second example could derive from fishing disturbance induced changes in pelagic primary production, in and of itself, influencing the community structure or abundance of vertebrate fauna which could affect the seabed OC downstream (Brown et al., 2010). Empirical studies which take a whole system approach to seabed OC may allow some of these interactions to be better understood in the future.

## 3 EXPERIMENTAL RESULTS

From a literature review (see Supporting Information), 38 peer‐reviewed studies were identified which investigated the impact of mobile demersal fishing on the seabed, and directly measured OC or organic matter (OM) and/or remineralization rates in seabed sediments (Table 1). The 38 studies covered 12 oceanic realms with greatest representation from the Northeast Atlantic (37%), Mediterranean (24%), and Northwest Atlantic (16%) (Table 1). The majority of studies (60%) investigated impacts of commercial fishing activities not under the direct control of the investigators. The remainder either used experimental trawling/dredging methods (34%) or a mixture of experimental trawling and monitoring of commercial fishing (5%) (Table 1). Studies which used experimental fishing methods generally considered acute disturbance events which were conducted by the investigators for periods ranging from 1 day to 15 months, but mostly lasting only a single day (Table S1). Commercial fishing studies generally consider more chronic impacts, comparing areas with different levels of fishing intensities or areas closed to mobile demersal fishing for periods of months to multiple years (Table S1). As evidence to support the different treatment levels, commercial fishing studies rely on information such as vessel monitoring data, fishing legislation or local environmental knowledge (Table S1).

**TABLE 1:** Summary of studies which investigated the impact of mobile demersal fishing on the seabed and directly measured organic carbon (OC) or organic matter (OM), and/or remineralization rates of OC/OM in the sediment. The last two columns indicate whether the presence or increase in demersal fishing activity was reported to cause lower (red), higher (green), no significant effect (orange) or mixed effects (grey) in the concentration or content of OC/OM (‘OC/OM’), or organic carbon remineralization rate (‘Remin’ rate’), within seabed sediments

Reference	Oceanic region	Sediment	Depth (m BCD)	Gear	Study type	Impact type	Sediment depth	Investigations	OC/OM	Remin' rate
Adriano et al. (2005)	N Mediterranean	Sandy‐mud	~1	Clam dredge	BA	Commercial fishing	Homog' surface	1		
Atkinson et al. (2011)	SE Atlantic	Muddy‐sand	346–459	Otter‐trawl	LH	Commercial fishing	Homog' surface	1		
Bhagirathan et al. (2010)	N Indian	Mud	15–40	Otter‐trawl	BA	Experimental	Homog' surface	1		
Brown et al. (2005)	NE Pacific	Muddy‐sand	25–35	Otter‐trawl	BACI IC	Experimental Commercial fishing	0–5 cm	2		
Dolmer et al. (2001)	NE Atlantic	Muddy‐sand	7	Mussel dredge	IC	Experimental	Homog’ surface	1		
Eleftheriou and Robertson (1992)	NE Atlantic	Sand	~7	Scallop dredge	BA	Experimental	0–6 cm	1		
Ferguson et al. (2020)	SW Pacific	Muddy‐sand	4	Otter trawl	BACI	Experimental	0.5 cm	1		
Fiordelmondo et al. (2003)	N Mediterranean	Sand	~2	Clam dredge	IC	Experimental	1 cm	1		
Goldberg et al. (2014)	NW Atlantic	Fine sand	3–5	Hydraulic dredge	IC	Experimental	~0–20 cm	1		
Hale et al. (2017)	NE Atlantic	Mud & Sand	19–29	Otter trawl & Scallop dredge	LH	Commercial fishing	1 cm	2		
Lamarque et al. (2021)	NE Atlantic	Sandy‐mud	33–78	Mixed trawls	LH	Commercial fishing	0–1 cm	1	*	
Lindeboom and de Groot (1998)	NE Atlantic	Mud & Sand	30–75	Mixed trawls	BACI IC	Experimental Commercial fishing	Homog’ surface/0–10 cm	3		
Liu et al. (2011)	W Pacific	Sandy‐mud	20	Mixed trawls	IC	Commercial fishing	Homog’ surface	1		
Martín, Puig, Masque, et al. (2014)	NW Mediterranean	Mud	453–591	Otter trawl	IC	Commercial fishing	0–50 cm	1		
Mayer et al. (1991)	NW Atlantic	Mud & Mixed	8–20	Otter trawl & Scallop dredge	IC	Experimental	0–12 cm	2		
McLaverty et al. (2020)	NE Atlantic	Sandy‐mud	3–11	Mussel dredge	LH	Commercial fishing	Homog’ surface	4	*	
Mercaldo‐Allen et al. (2016)	NW Atlantic	Fine sand	3–5	Hydraulic dredge	IC	Experimental	Homog’ surface	1		
Meseck et al. (2014)	NW Atlantic	Fine sand	5–6	Hydraulic dredge	BACI	Experimental	~0–20 cm	1		
Morys et al. (2021)	Baltic	Muddy‐sand	12	Benthic Dredge	IC	Experimental	0–15 cm	1		
Palanques et al. (2014)	NW Mediterranean	Mud	40–70	Otter trawl	IC	Commercial fishing	0–30 cm	1		
Paradis et al. (2019)	SW Mediterranean	Mud	550	Otter trawl	IC	Commercial fishing	0–35 cm	1		
Paradis et al. (2021)	NW Mediterranean	Mud	425–494	Otter trawl	IC	Commercial fishing	0–10 cm	1		
Polymenakou et al. (2005)	NE Mediterranean	Sandy‐mud	30–51	Otter trawl	BA	Commercial fishing	0–1 cm	1		
Pusceddu et al. (2005a)	NE Mediterranean	Sandy‐mud	30–80	Otter trawl	BA	Commercial fishing	0–10 cm	1		
Pusceddu et al. (2014)	NW Mediterranean	Mud	454–556	Otter trawl	IC	Commercial fishing	0–10 cm	1		
Rajesh et al. (2019)	N Indian	Sand	5–35	Beam trawl	BA	Experimental	Homog’ surface	2		
Ramalho et al. (2018)	NE Atlantic	Muddy‐sand	285–550	Otter trawl	IC	Commercial fishing	Homog’ surface	1		
Ramalho et al. (2020)	NE Atlantic	Muddy‐sand	285–550	Otter trawl	LH	Commercial fishing	0–5 cm	1		
Rosli et al. (2016)	SW Pacific	Sandy‐mud	670–1561	Otter trawl	LH	Commercial fishing	0–1 cm	2	*	
Sciberras et al. (2016)	NE Atlantic	Mud & Sand	20–43	Otter trawl & Scallop dredge	LH	Commercial fishing	Homog’ surface	2		
Serpetti et al. (2013)	NE Atlantic	Muddy‐sand	769–823	Mixed trawls	IC	Commercial fishing	0–10 cm	1		
Sheridan and Doerr (2005)	NW Atlantic	Mud & Sand	5–20	Otter trawl	IC	Commercial fishing	0–5 cm	1		
Smith (2000)	NE Mediterranean	Sandy‐mud	~200	Otter trawl	BACI	Commercial fishing	0–4 cm	1		
Tiano et al. (2019)	NE Atlantic	Muddy‐sand	34	Mixed trawls	BA	Experimental	0–2.5 cm	2		
Trimmer et al. (2005)	NE Atlantic	Muddy‐sand	~20–80	Beam trawl	LH	Commercial fishing	0–10 cm	2	*	
van de Velde et al. (2018)	NE Atlantic	Mud	~7	Unknown	BA	Commercial fishing	0–30 cm	1		
Wang et al. (2021)	W Pacific	Mud & Sand	1–28	Mixed trawls	Recovery	Commercial fishing	Homog’ surface	1		
Watling et al. (2001)	NW Atlantic	Muddy‐sand	15	Scallop dredge	BA	Experimental	0–15 cm	1	*	

Notes

For ‘Study type’: BA = Before–after fishing impact, IC = Impact–control site comparison, LH = low to high impacted sites, BACI = before–after control–impact, ‘Recovery’ = change after removal of commercial fishing. ‘Investigations’ = the number of individual investigations conducted in each study. ‘Homog’ surface’ = A homogenized sample of surface sediment was measured (often taken from a grab sample). ‘BCD’ = Below chart datum. For ‘OC/OM’, those with an asterisk (*) indicate where further analysis was needed—see Supporting Information. The ‘OC/OM’ column is empty for Polymenakou et al. (2005) as the result was based on the same data which are reported in Pusceddu et al. (2005a).

A variety of experimental set‐ups were employed including impact–control site comparisons (39%), before–after fishing impact (24%) and low–high impact contrasts which lacked controls (21%). Additionally, 13% of studies used a before–after control–impact design either alone or in combination with an impact–control experiment, and one investigated the recovery of seabed sediment OC after a long‐term closure to mobile demersal fishing (Table 1). It should be noted that for many of these studies, in areas considered ‘control sites’, there is the potential for them to still be affected by mobile demersal fishing activities. This often occurs due to insufficient monitoring (e.g. no vessel monitoring system data on smaller vessels), lack of enforcement (i.e. within a supposed closed area) or lack of recovery time since cessation of fishing given the long timescales of recovery for many habitats (Roberts, 2007).

Of the 38 studies identified, 10 investigated the effect of mobile demersal fishing across multiple sites, habitat types or gear types and made inferences for each investigation separately (Table 1), producing a total of 51 individual investigations (Table S1). Most of these considered the impact of demersal trawling gears (65%) with the remainder assessing the impact from types of dredge fisheries (35%) (Table S1). The majority of investigations only investigated the impact of mobile demersal fishing on OC in homogenized surface samples (35%) or in sediment depths to a maximum of 5 cm (29%) (Table S1). Of the remaining investigations, most considered sediment depths up to a maximum of 20 cm (28% of all investigations). Only 6% of investigations measured impacts up to 35 cm and only one up to 50 cm (Table S1). The depth of the seabed under investigation ranged from 1 to 1561 m BCD (below chart datum); however, the majority of investigations (67%) were conducted below 50 m (Table S1).

There were only seven inferences regarding the impact of mobile demersal fishing pressure on in situ seabed sediment carbon remineralization rates. Of these, four reported that demersal fishing activity decreased remineralization rate in seabed sediments, with three concluding the opposite (Table S1). Although no clear trend was identified between studies, one hypothesis is that the direction of effects may be dependent on local hydrographic conditions. For example, in more depositional environments, mobile demersal fishing may cause oxygenation of sediments and redeposition of recently expulsed organic material back to the seabed, leading to an increase in remineralization rate (Duplisea et al., 2001; Polymenakou et al., 2005; van de Velde et al., 2018). In more hydrologically active environments, resuspension and lateral/vertical transport of sediments may be expected to reduce OC in surface sediments which, along with removal of fauna, could limit the rate of remineralization (De Borger et al., 2021; Morys et al., 2021; Pusceddu et al., 2014; Tiano et al., 2019).

**FIGURE 2:**
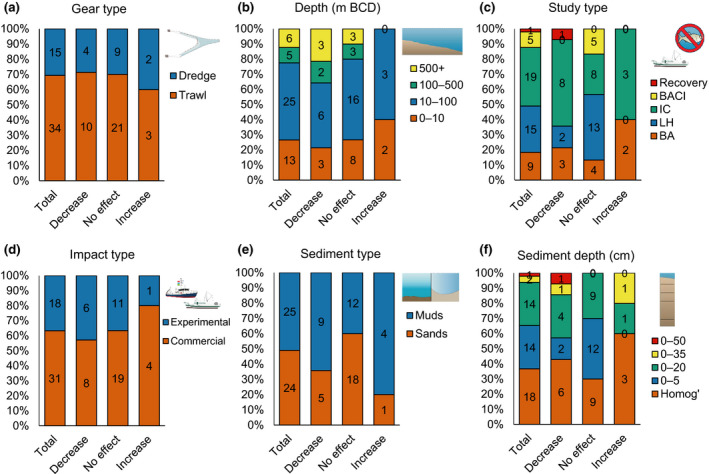
Environmental and experimental characteristics of investigations assessing the effect of mobile demersal fishing on organic carbon (OC). Bar charts represent the proportion of investigations for each category, with inset numbers indicating frequency. Data are shown for all investigations which directly measured changes of OC/OM (organic carbon/organic matter) concentration/content in seabed sediments (‘Total’, *n* = 49), those which reported a decrease in OC/OM due to mobile demersal fishing (‘Decrease’, *n* = 14), investigations with no significant effect (‘No effect’, *n* = 30) and those which found an increase in OC/OM due to mobile demersal fishing (‘Increase’, *n* = 5). ‘Homog’ = homogenized surface sediment. BCD = Below chart datum. Symbols courtesy of Integration and Application Network (ian.umces.edu/media‐library)

Of the 51 individual investigations, 49 measured changes in OC/OM concentration/content. A finding of no significant effect was reported in 61% of investigations; 29% reported lower OC in fished sites compared to unfished control sites or in areas with higher fishing intensities; with the remaining 10% of investigations reporting higher OC (Table S1). Patterns in the environmental and experimental characteristics between different outcomes were largely indistinct (Figure 2). The median depth at which the research was conducted was relatively similar between different experimental outcomes, although those that reported an increase in OC were generally conducted at shallower depths (Figure 2b, Table S1). Median depths for the experimental outcomes were 22, 31 and 20, and ranged between 2–591, 4–1561 and 1–55 m for studies which reported a decrease, no significant effect and an increase in OC respectively (Table S1). Those investigations which reported no significant effect of demersal fishing on OC had some distinguishing features: They were more likely to be undertaken on sand, measure OC to shallower sediment depths and use a study design comparing sites with different levels of fishing intensities but lacking controls (Figure 2). All these factors may make it more challenging to detect impact signatures from mobile demersal fishing, especially as sandy sediments are generally characterized by low quantities of OC, higher levels of natural disturbance, higher oxygen penetration depths and faster remineralization rates when compared to muds (Burdige, 2007; Huettel et al., 2014).

While this literature review gives a summary of empirical research and an indication into patterns and drivers of experimental outcomes, it is not exhaustive so cannot be considered fully systematic. For example, it only considered peer‐reviewed primary literature obtainable from two bibliographic databases and ignored grey literature. Additionally, we made no attempt to critically assess the quality or validity of each study before its inclusion, and the data extracted were largely qualitative or semiquantitative in nature. A more thorough systematic review and meta‐analysis may provide further evidence and/or varying results.

## 4 FUTURE RESEARCH

As highlighted by the varied experimental results, there is a clear need for further research into the potential impact of mobile demersal fishing on OC burial and long‐term storage in seabed sediments under different environmental settings. Recent first‐order estimates have suggested that globally, mobile demersal fishing could remineralize between 0.16 and 0.4 Gt of OC from marine sediment stores annually (Sala et al., 2021). It has also been suggested that historical trawling on global continental slopes could have removed ~0.06 Gt of OC from the uppermost centimetre of sediment alone (Paradis et al., 2021). In addition, it has been estimated that ~0.002 Gt of OC is remineralized from UK shelf sediments each year by mobile demersal fishing (Luisetti et al., 2019). Although these estimates contain large uncertainties, their scale reveals the large potential for mobile demersal fishing to reduce carbon stores.

Following disturbance and/or resuspension by mobile demersal fishing, a proportion of OC may become remineralized in the seabed or in the water column due to the processes discussed above; however, some will simply remain in situ and be reburied, and a further proportion will be transported over a range of distances eventually being consumed or reburied (Lovelock et al., 2017; Pendleton et al., 2012). A key research gap is the tracking and quantification of OC that follows each of these processes in different environmental settings, in areas with different sediment carbon characteristics and under different types of fishing impact. Sala et al. (2021) only account for remineralization of disturbed OC which remains in situ or resettles within 1 km^2^, as they consider the fate of sediment which stays in suspension as unknown. In their paper, Sala et al. (2021) consider that 87% of disturbed OC remains in situ or resettles uniformly across global fishing effort, and of this anything between 1 and 69.3% will be remineralized. Their estimate is based on a simple model including (1) an estimate of the proportion of OC which is labile and (2) an average first‐order reaction constant. For both model parameters, they used basin‐scale average values for the incoming OC flux rather than values representative for the sedimentary stock which are much lower (Arndt et al., 2013; Soetaert et al., 1996), with the result that the impact of fishing may have been overestimated. In their regional study, Luisetti et al. (2019) use an upper estimate that 100% of the OC resuspended by mobile demersal fishing will be remineralized, but do not consider the fate of OC that is disturbed but remains in situ.

Although the studies by Sala et al. (2021) and Luisetti et al. (2019) give a representation to the scale of OC which may be lost, improved quantification of these metrics is clearly needed before accurate measures of OC lost, or inorganic carbon produced, can be quantified. OC in seabed sediments is not naturally inert and only a small percentage of OC that reaches the seabed is stored with the remainder passing through a range of aerobic and anaerobic remineralization pathways to varying sediment depths (Arndt et al., 2013; Burdige, 2007; Middelburg, 2018). Additionally, the characteristics of OC in seabed sediments are highly heterogeneous, with numerous chemical compounds and both abiotic and biotic environmental settings all influencing OC reactivity (Arndt et al., 2013; LaRowe et al., 2020; Middelburg, 2018). Thus, more consideration is needed to understand the influence of natural remineralization rates and the vulnerability of seabed OC to remineralization under different environmental settings, and therefore how to quantify the additional effect of mobile demersal fishing in each area. In seabed sediment habitats with high hydrodynamic activity, low deposition rates, large oxygen penetration depths and highly refractory OC, the effect of disturbance by demersal fishing on OC may be limited.

The cumulative or finite nature of disturbance by demersal mobile fishing on OC stores must also be considered, and currently, it is not clear how much of the estimated 360 Gt of OC in the top 1 m of sediment is actually threated by the activity (Atwood et al., 2020). While mobile demersal fishing can only penetrate between around 2 and 20 cm into the sediment (Hiddink et al., 2017), repeated chronic impacts may continue to disturb and displace sediment more deeply (Sala et al., 2021). It is also possible that in chronically fished areas, significant further loss of OC stores will not occur due to historic depletion in surface OC stocks (Sala et al., 2021). By contrast, if new fishing grounds emerge (Gogarty et al., 2020; e.g. Morato et al., 2006), this could lead to large OC stocks potentially becoming vulnerable to remineralization.

The scale of penetration and legacy of disturbance must also be considered alongside differing sediment depth OC profiles (Martín, Puig, Masque, et al., 2014; Middelburg, 2018; Paradis et al., 2019). In the study by Sala et al. (2021), it is assumed that carbon stocks are equally distributed in the top metre of sediment; however in the vast majority of cases, it is known that this does not occur (Berner, 1982; Burdige, 2007). In stable accreting sediments, OC concentrations are generally highest at the surface and reduce with depth until a steady‐state burial rate is reached (Arndt et al., 2013; Burdige, 2007). Sediments which are frequently mixed either through natural or anthropogenic disturbances may have more uniform OC depth profiles (Dauwe & Middelburg, 1998; Martín, Puig, Masque, et al., 2014; Middelburg, 2018; Paradis et al., 2019); however, historical signatures of OC accumulation, disturbance or deposits may also be identified under surface layers of accreting or mixed sediments dependent on the depth limit of investigations and the geology of the site (de Haas et al., 2002; Martín et al., 2008; Palanques et al., 2014). The majority of studies identified in this review investigated the impact of OC in homogenized surface sediments or to sediment layers only up to 5 cm (Table S1). It is likely that in certain environmental settings, disturbance signatures could be identified much deeper within the sediment and should be better considered (Martín, Puig, Masque, et al., 2014). As the characteristics and reactivity of OC can also alter with sediment depth (Arndt et al., 2013; LaRowe et al., 2020; Middelburg, 2018), it is paramount that future studies consider the effects of mobile demersal fishing on a range of sediment layers. The scale and direction of effects of mobile demersal fishing disturbance on surface sediment OC may differ from that in deeper layers.

There is also a need to identify a clear baseline from which changes in OC can be measured. Standing stock of OC in global seabed sediments is relatively well resolved at a number of spatial scales (Atwood et al., 2020; Diesing et al., 2017, 2021; Lee et al., 2019; Legge et al., 2020; Luisetti et al., 2019; e.g. Seiter et al., 2004; Smeaton et al., 2021). However, precise estimates of OC remineralization, accumulation and burial rates are generally lacking (Berner, 1982; Burdige, 2007; Diesing et al., 2021; Keil, 2017; Legge et al., 2020; Luisetti et al., 2019; Wilkinson et al., 2018). For robust conclusions to be drawn studies which aim to quantify the impact of demersal fishing on carbon storage must therefore quantify both before and after scenarios.

On land, retrospective analyses of changes in human use and vegetation cover have been critical to estimating how people have altered the planetary carbon cycle. It is vital that this historical context is also considered when further investigating the potential impact of mobile demersal fishing on global seabed OC storage, and the opportunities for recovery if this pressure is removed. Due to the extended timeframes needed for some seabed habitats to fully recover, true long‐term protection and monitoring of OC are needed to fully deduce carbon storage potential. Without considering areas of seabed that have experienced genuine long‐term protection, it is not possible to gain an accurate baseline from which impacts can be compared (Pinnegar & Engelhard, 2008). Within this review, we identified only one study which looked at the direct recovery of OC in seabed sediments following the medium‐ to long‐term removal of fishing pressure (Wang et al., 2021). Gaining further evidence of this nature is vital to understand how much OC can accumulate when mobile demersal fishing is removed, and how this may change over the course of recovery.

It is important that future research into the impact of mobile demersal fishing on carbon storage is focused in areas which are expected to contain significant stocks of OC or have large future burial potential, based on their geography (Atwood et al., 2020), sediment characteristics (Smeaton et al., 2021) and local hydrology (Lee et al., 2019). Research should also focus on areas that overlap with significant mobile demersal fishing pressure (Amoroso et al., 2018; Kroodsma et al., 2018; Sala et al., 2021), and where this can be compared to areas that could be considered truly ‘unfished’, either from well‐enforced protected areas or specific environmental settings.

## 5 CONCLUDING REMARKS

Seabed sediments are one of the planet's primary OC stores and strongly influence the oceanic sink for atmospheric CO_2_ (Atwood et al., 2020; Gruber et al., 2019; Sala et al., 2021; Watson et al., 2020). It is an urgent priority to better understand the effect of mobile fishing gear use on seabed OC storage, and to incorporate clear blue carbon considerations into global seabed management. As only around 2%–3% of the world's seabed is currently closed to trawling and dredging (Marine Conservation Institute, 2021; Roberts et al., 2017), increasing the scale of protection could offer huge climate change mitigation potential and bring corresponding gains in biodiversity (Roberts et al., 2017, 2020; Sala et al., 2021; Seddon et al., 2019). Across the world, mobile demersal fisheries are highly fuel inefficient and produce most of the fishing industry's direct greenhouse gas emissions (Parker et al., 2018). A shift to less damaging fishing methods could provide major net benefits for increasing natural carbon storage in the seabed, whilst significantly reducing emissions of CO_2_.

The results of recent regional and global scale publications which have calculated first‐order estimates of CO_2_ produced from disturbance to seabed sediments by mobile demersal fishing must be viewed with both concern and caution (Luisetti et al., 2019; Paradis et al., 2021; Sala et al., 2021). As identified in this review, demersal fishing by trawling and dredging is in some cases likely to limit the storage of OC, but to draw firm conclusions more experimental and modelling studies that cover a wide range of environmental settings, habitat types and fishing pressures are required to address the large number of unknowns and site‐specific drivers associated with the status of OC on the seabed.

## CONFLICTS OF INTEREST

The authors declare that they have no conflict of interest.

## AUTHOR CONTRIBUTIONS

GE prepared the original draft and undertook the literature review. CR & CN contributed to conceptualization and planning. All authors contributed to writing, reviewing and editing.

## Supporting information

Table S1

Table S2

## Data Availability

The data that support the findings of this study are available in the Supporting Information of this article or are openly available in figshare at https://doi.org/10.6084/m9.figshare.16776250.
