# Supplementary material for: The impact of mobile demersal fishing on carbon storage in seabed sediments
Source: Glob Chang Biol. 2022 Feb 17;28(9):2875–94. doi: 10.1111/gcb.16105 (PMC9307015; doi:10.1111/gcb.16105)
Supplement: Supplementary file 2 — Table S2 [file GCB-28-2875-s002.docx]

Supporting Information

The impact of mobile demersal fishing on carbon storage in seabed sediments

_Graham Epstein, Jack J. Middelburg, Julie P. Hawkins, Catrin R. Norris, Callum M. Roberts_

**Details of literature review**

**Article search**

Literature searches were conducted in April 2021 in Web of Science and Scopus. Within Web of Science, its “Core collection” was searched via the field “Topic”, which examines a paper’s title, abstract, author, keywords and “keywords plus”. Within Scopus, the “Advanced search” was run via the field “Title-Abs-Key”, which scans a paper’s title, abstract and keywords. Within both databases the same search string was used:

*(Sediment* OR Mud* OR Sand* OR Clay* OR Silt* OR Gravel*) AND (Coast* OR Sea* OR Ocean* OR Estur* OR Estuary OR Marine) AND (Trawl* OR Dredg* OR “Demersal Fishing” OR “Demersal Fisher*” OR “Bottom Fishing” OR “Bottom Fisher*” OR “Benthic Fishing” OR “Benthic Fisher*”) AND (“Organic Carbon” OR “Organic Matter” OR “Organic Content” OR “Blue Carbon” OR Remineralisation OR Remineralization OR “Carbon mineralisation” OR “Carbon mineralization”)*

In addition, a bibliographic search of all relevant articles identified was conducted to ensure all appropriate articles were included. Only English language articles were assessed.

**Inclusion criteria**

To be included in this review studies were required to meet the following characteristics:

- *Habitat -* Any subtidal benthic marine environment, including estuarine; but not typical vegetated blue carbon habitats (saltmarsh, seagrass and mangroves).
- *Fishing pressure* - Included studies had to investigate or quantify demersal mobile fishing pressure (i.e. benthic trawling or dredging for fish or shellfish).
- *Sampling -* Seabed sediments must have been sampled (or modelled) to be included in the review. Studies which only sampled the water column or biota were excluded during screening.
- *Response metric -* Levels of organic carbon (OC), organic matter (OM) or carbon remineralisation rates had to be sampled within the seabed, or used directly as a response variable within each study
- *Interaction -* The presence or magnitude of mobile demersal fishing pressure needed to be related to the response variable of interest in some way. This may have been conducted using a variety of methods such as Before-After impact studies, Impact-Control site comparisons, Before-After removal of pressure, or measures across gradients of pressure. Studies that contained no contrasts in levels of fishing pressure were excluded.

**Screening process**

All articles identified from the searches were exported into a single EndNote library then duplicates removed. Screening was conducted via a hierarchical process that first assessed title, then abstract and finally full text. At each stage an article was assessed against the inclusion criteria described above, with those considered relevant or of unclear relevance passing to the next level of assessment. Once relevant articles were identified, all their bibliographic references were exported to a new EndNote folder by selecting all “Related Records” for each entry in Web of Science. The screening process was then conducted for a second round.

In total, 1,124 articles were identified from the search, of which 367 were duplicates. Removal of the latter lead to 757 screened with 694 excluded at the title and abstract stages. Of the 63 articles assessed at full text, 32 were considered relevant. The reasons why the other 31 were excluded were as follows: one full text was not available; one study consisted of re-analysis and republication of data from a previous study; one study was about seagrass; eight did not consider mobile demersal fishing; six had no direct measurements of seabed sediments; seven did not quantify OC/OM or carbon remineralisation; and seven studies had no contrasts between levels of fishing impact. The second round of screening, which considered all cited literature within the 34 relevant studies, identified a further 4 relevant studies for inclusion. Finally, all authors reviewed the list of identified studies and contributed known relevant papers that were absent or very recently published – adding a further 2 studies.

**Table S2.** Studies excluded at full text and reasons for their exclusion. See below for full reference of each study.

| **Study** | **Reason for exclusion** |
| --- | --- |
| Boudaya, Mosbahi, Dauvin, and Neifar (2019) | No contrast between levels of fishing impact. |
| Cesar and Frid (2009) | Does not consider mobile demersal fishing (hand raked bivalve fishing). |
| Coughlan et al. (2015) | Did not quantify OC/OM/remineralisation |
| Daly et al. (2018) | No direct measurement of seabed sediments (pelagic sampling) |
| De Borger, Tiano, Braeckman, Rijnsdorp, and Soetaert (2021) | No direct measurements of seabed sediments (modelling study). |
| De Haas, Boer, and Van Weering (1997) | No contrasts between levels of fishing impact. |
| Dernie, Kaiser, and Warwick (2003) | Does not consider mobile demersal fishing (intertidal digging). |
| Dounas et al. (2007) | No direct measurement of seabed sediments (pelagic sampling) |
| Dounas, Davies, Hayes, Arvanitidis, and Koulouri (2005) | Full text unavailable. |
| Duplisea, Jennings, Malcolm, Parker, and Sivyer (2001) | No direct measurements of seabed sediments (modelling study). |
| Durrieu de Madron et al. (2020) | No contrast between levels of fishing impact. |
| Gonzalez-Correa et al. (2005) | Seagrass study. |
| Gonzalez-Irusta, Punzon, and Serrano (2012) | No contrasts between levels of fishing impact. |
| Holmer, Ahrensberg, and Jorgensen (2003) | Did not quantify OC/OM/remineralisation |
| Jaleel et al. (2015) | No contrasts between levels of fishing impact. |
| Lampadariou, Hatziyanni, and Tselepides (2005) | Re-analysis of data from previous study for different hypothesis. |
| Larsen, Thamdrup, Shimmield, and Glud (2013) | No contrasts between levels of fishing impact. |
| Lu et al. (2020) | Does not consider mobile demersal fishing. |
| Mattos and de Almeida (2016) | Does not consider mobile demersal fishing. |
| Mil-Homens et al. (2014) | Does not consider mobile demersal fishing. |
| Nayar, Miller, Hunt, Goh, and Chou (2007) | Does not consider mobile demersal fishing. |
| Pusceddu, Bianchelli, and Danovaro (2015) | No direct measurement of seabed sediments (pelagic sampling) |
| Rijnsdorp et al. (2016) | No direct measurements of seabed sediments |
| Roman et al. (2016) | No contrasts between levels of fishing impact. |
| Romano et al. (2016) | Did not quantify OC/OM/remineralisation. |
| Sane, Martin, Puig, and Palanques (2013) | Did not quantify OC/OM/remineralisation. |
| Schwinghamer et al. (1998) | Did not quantify OC/OM/remineralisation. |
| Sigamani, Perumal, Arumugam, Preetha Mini Jose, and Veeraiyan (2015) | Does not consider mobile demersal fishing. |
| Viaroli, Bartoli, Giordani, Azzoni, and Nizzoli (2003) | Does not consider mobile demersal fishing (hand raked bivalve fishing). |
| Warnken et al. (2003) | Did not quantify OC/OM/remineralisation. |
| Watling (2014) | Did not quantify OC/OM/remineralisation. |

**Data extraction and analyses**

If individual studies investigated the effect of mobile demersal fishing across multiple sites, habitat types, or gear-types, and made inferences for each investigation separately, these were separated into individual experimental contrasts for discussion within this review. This led to 62 different experimental contrasts identified across the 40 studies examined (Table S1). Qualitative or semi-quantitative descriptive information extracted from each relevant article included the study location, sediment type, water depth, fishing gear type, study design, method for measuring fishing impact, impact duration (or period for which data were available), depth within the sediment to which sampling was conducted, and the frequency of any temporal sampling (see Table S1 for further details). If information on sediment type or the fishing gear was lacking, a grey literature search was conducted into the study-site and/or local fishery to obtain a qualitative description of these characteristics.

In 35 of the 40 studies examined, direct inference on the significance and direction of effects of mobile demersal fishing on OC/OM was made within the publication. This was identified through the display of formal statistical tests or clear statements made within the text. In the five remaining studies, no direct inference was made within the text or supplementary information, however applicable data were presented. In these cases additional analysis was required to infer the impact of demersal mobile fishing on OC/OM (Table S1; Epstein, 2021). In these cases, basic statistical tests were conducted on the data presented within the publication to identify the likely significance and direction of effect. All statistical analyses were carried out in R 4.0.4. Studies which collected data on OC/OM across a continuous gradient of fishing pressure were assessed using Spearman rank correlations, with associated p-values calculated using the *cor.test* function in base R. Those studies which considered OC/OM levels across discrete gradients of pressure were analysed with basic linear models.

**References**

Boudaya, L., Mosbahi, N., Dauvin, J. C., & Neifar, L. (2019). Structure of the benthic macrofauna of an anthropogenic influenced area: Skhira bay (gulf of gabès, central mediterranean sea). Environmental Science and Pollution Research, 26(13), 13522-13538. doi:10.1007/s11356-019-04809-8

Cesar, C. P., & Frid, C. L. J. (2009). Effects of experimental small-scale cockle (Cerastoderma edule L.) fishing on ecosystem function. Marine Ecology-an Evolutionary Perspective, 30, 123-137. doi:10.1111/j.1439-0485.2009.00305.x

Coughlan, M., Wheeler, A. J., Dorschel, B., Lordan, C., Boer, W., van Gaever, P., . . . Morz, T. (2015). Record of anthropogenic impact on the Western Irish Sea mud belt. Anthropocene, 9, 56-69. doi:10.1016/j.ancene.2015.06.001

Daly, E., Johnson, M. P., Wilson, A. M., Gerritsen, H. D., Kiriakoulakis, K., Allcock, A. L., & White, M. (2018). Bottom trawling at Whittard Canyon: Evidence for seabed modification, trawl plumes and food source heterogeneity. Progress in Oceanography, 169, 227-240. doi:10.1016/j.pocean.2017.12.010

De Borger, E., Tiano, J., Braeckman, U., Rijnsdorp, A. D., & Soetaert, K. (2021). Impact of bottom trawling on sediment biogeochemistry: a modelling approach. Biogeosciences, 18(8), 2539-2557. doi:10.5194/bg-18-2539-2021

De Haas, H., Boer, W., & Van Weering, T. C. E. (1997). Recent sedimentation and organic carbon burial in a shelf sea: The North Sea. Marine Geology, 144(1-3), 131-146. doi:10.1016/S0025-3227(97)00082-0

Dernie, K. M., Kaiser, M. J., & Warwick, R. M. (2003). Recovery rates of benthic communities following physical disturbance. Journal of Animal Ecology, 72(6), 1043-1056. doi:10.1046/j.1365-2656.2003.00775.x

Dounas, C., Davies, I., Triantafyllou, G., Koulouri, P., Petihakis, G., Arvanitidis, C., Sourlatzis, G., Eleftheriou, A. (2007). Large-scale impacts of bottom trawling on shelf primary productivity. Continental Shelf Research, 27(17), 2198-2210. doi:10.1016/j.csr.2007.05.006

Dounas, C., Davies, I. M., Hayes, P. J., Arvanitidis, C. D., & Koulouri, P. T. (2005). The effect of different types of otter trawl ground rope on benthic nutrient releases and sediment biogeochemistry. In B. W. Barnes & J. P. Thomas (Eds.), Benthic Habitats and the Effects of Fishing (Vol. 41, pp. 539-544). Bethesda: Amer Fisheries Soc.

Duplisea, D. E., Jennings, S., Malcolm, S. J., Parker, R., & Sivyer, D. B. (2001). Modelling potential impacts of bottom trawl fisheries on soft sediment biogeochemistry in the North Sea. Geochemical Transactions, 2, 112-117. doi:10.1039/b108342b

Durrieu de Madron, X., Stabholz, M., Heimbürger-Boavida, L. E., Aubert, D., Kerherve, P., Charriere, B., & Ludwig, W. (2020). Approaches to evaluate spatial and temporal variability of deep marine sediment characteristics under the impact of dense water formation events. Mediterranean Marine Science, 21(3), 527-544. doi:10.12681/MMS.22581

Epstein, G. (2021), Additional analysis on the potential for mobile demersal fishing to reduce carbon storage and sequestration in seabed sediments, FigShare, Dataset, <https://doi.org/10.6084/m9.figshare.16776250>

Gonzalez-Correa, J. M., Bayle, J. T., Sanchez-Lizasa, J. L., Valle, C., Sanchez-Jerez, P., & Ruiz, J. M. (2005). Recovery of deep Posidonia oceanica meadows degraded by trawling. Journal of Experimental Marine Biology and Ecology, 320(1), 65-76. doi:10.1016/j.jembe.2004.12.032

Gonzalez-Irusta, J. M., Punzon, A., & Serrano, A. (2012). Environmental and fisheries effects on Gracilechinus acutus (Echinodermata: Echinoidea) distribution: is it a suitable bioindicator of trawling disturbance? Ices Journal of Marine Science, 69(8), 1457-1465. doi:10.1093/icesjms/fss102

Holmer, M., Ahrensberg, N., & Jorgensen, N. P. (2003). IMPACTS OF MUSSEL DREDGING ON SEDIMENT PHOSPHORUS DYNAMICS IN A EUTROPHIC DANISH FJORD. Chemistry and Ecology, 19(5), 343-361. doi:10.1080/02757540310001596708

Jaleel, K. U. A., Parameswaran, U. V., Gopal, A., Khader, C., Ganesh, T., Sanjeevan, V. N., . . . Gupta, G. V. M. (2015). Evaluation of changes in macrobenthic standing stock and polychaete community structure along the south eastern Arabian Sea shelf during the monsoon trawl-ban. Continental Shelf Research, 102, 9-18. doi:10.1016/j.csr.2015.04.011

Lampadariou, N., Hatziyanni, E., & Tselepides, A. (2005). Meiofaunal community structure in Thermaikos Gulf: Response to intense trawling pressure. Continental Shelf Research, 25(19-20), 2554-2569. doi:10.1016/j.csr.2005.08.016

Larsen, M., Thamdrup, B., Shimmield, T., & Glud, R. N. (2013). Benthic mineralization and solute exchange on a Celtic Sea sand-bank (Jones Bank). Progress in Oceanography, 117, 64-75. doi:10.1016/j.pocean.2013.06.010

Lu, X., Zhou, F., Chen, F., Lao, Q., Zhu, Q., Meng, Y., & Chen, C. (2020). Spatial and seasonal variations of sedimentary organic matter in a subtropical bay: Implication for human interventions. International Journal of Environmental Research and Public Health, 17(4). doi:10.3390/ijerph17041362

Mattos, P. R., & de Almeida, T. C. M. (2016). Spatiotemporal distribution of the benthic macrofauna in an urbanized subtropical estuary: Environmental variations and anthropogenic impacts. Brazilian Journal of Oceanography, 64(3), 227-238. doi:10.1590/S1679-87592016111306403

Mil-Homens, M., Vale, C., Raimundo, J., Pereira, P., Brito, P., & Caetano, M. (2014). Major factors influencing the elemental composition of surface estuarine sediments: The case of 15 estuaries in Portugal. Marine Pollution Bulletin, 84(1-2), 135-146. doi:10.1016/j.marpolbul.2014.05.026

Nayar, S., Miller, D. J., Hunt, A., Goh, B. P. L., & Chou, L. M. (2007). Environmental effects of dredging on sediment nutrients, carbon and granulometry in a tropical estuary. Environmental Monitoring and Assessment, 127(1-3), 1-13. doi:10.1007/s10661-006-9253-2

Pusceddu, A., Bianchelli, S., & Danovaro, R. (2015). Quantity and biochemical composition of particulate organic matter in a highly trawled area (thermaikos gulf, eastern mediterranean sea). Advances in Oceanography and Limnology, 6(1-2), 21-32. doi:10.4081/aiol.2015.5448

Rijnsdorp, A. D., Bastardie, F., Bolam, S. G., Buhl-Mortensen, L., Eigaard, O. R., Hamon, K. G., . . . Zengin, M. (2016). Towards a framework for the quantitative assessment of trawling impact on the seabed and benthic ecosystem. Ices Journal of Marine Science, 73, 127-138. doi:10.1093/icesjms/fsv207

Roman, S., Vanreusel, A., Romano, C., Ingels, J., Puig, P., Company, J. B., & Martin, D. (2016). High spatiotemporal variability in meiofaunal assemblages in Blanes Canyon (NW Mediterranean) subject to anthropogenic and natural disturbances. Deep-Sea Research Part I-Oceanographic Research Papers, 117, 70-83. doi:10.1016/j.dsr.2016.10.004

Romano, C., Fanelli, E., D'Anna, G., Pipitone, C., Vizzini, S., Mazzola, A., & Badalamenti, F. (2016). Spatial variability of soft-bottom macrobenthic communities in northern Sicily (Western Mediterranean): Contrasting trawled vs. untrawled areas. Marine Environmental Research, 122, 113-125. doi:10.1016/j.marenvres.2016.10.002

Sane, E., Martin, J., Puig, P., & Palanques, A. (2013). Organic biomarkers in deep-sea regions affected by bottom trawling: pigments, fatty acids, amino acids and carbohydrates in surface sediments from the La Fonera (Palamos) Canyon, NW Mediterranean Sea. Biogeosciences, 10(12), 8093-8108. doi:10.5194/bg-10-8093-2013

Schwinghamer, P., Gordon, D. C., Rowell, T. W., Prena, J., McKeown, D. L., Sonnichsen, G., & Guigne, J. Y. (1998). Effects of experimental otter trawling on surficial sediment properties of a sandy-bottom ecosystem on the Grand Banks of Newfoundland. Conservation Biology, 12(6), 1215-1222. doi:10.1046/j.1523-1739.1998.0120061215.x

Sigamani, S., Perumal, M., Arumugam, S., Preetha Mini Jose, H. M., & Veeraiyan, B. (2015). AMBI indices and multivariate approach to assess the ecological health of Vellar-Coleroon estuarine system undergoing various human activities. Marine Pollution Bulletin, 100(1), 334-343. doi:10.1016/j.marpolbul.2015.08.028

Viaroli, P., Bartoli, M., Giordani, G., Azzoni, R., & Nizzoli, D. (2003). Short term changes of benthic fluxes during clam harvesting in a coastal lagoon (Sacca di Goro, Po River Delta). Chemistry and Ecology, 19(2-3), 189-206. doi:10.1080/0275754031000119933

Warnken, K. W., Gill, G. A., Dellapenna, T. M., Lehman, R. D., Harper, D. E., & Allison, M. A. (2003). The effects of shrimp trawling on sediment oxygen consumption and the fluxes of trace metals and nutrients from estuarine sediments. Estuarine Coastal and Shelf Science, 57(1-2), 25-42. doi:10.1016/s0272-7714(02)00316-5

Watling, L. (2014). Trawling exerts big impacts on small beasts. Proceedings of the National Academy of Sciences of the United States of America, 111(24), 8704-8705. doi:10.1073/pnas.1407305111
